# Multisystemic effects of nanoplastics in immature rats subjected to prolonged oral exposure with particular emphasis on prooxidant potential

**DOI:** 10.3389/ftox.2026.1976947

**Published:** 2026-10-20

**Authors:** Kamil Adamiak, Grzegorz Sulkowski, Beata Dąbrowska-Bouta, Kamil Synoradzki, Małgorzata Frontczak-Baniewicz, Lidia Strużyńska

**Affiliations:** 1 Laboratory of Pathoneurochemistry, Department of Neurochemistry, Mossakowski Medical Research Institute Polish Academy of Sciences, Warsaw, Poland; 2 Doctoral School of Translational Medicine, Centre of Postgraduate Medical Education, Warsaw, Poland; 3 Environmental Laboratory of Pharmacological and Toxicological Research, Mossakowski Medical Research Institute Polish Academy of Sciences, Warsaw, Poland; 4 Clinical and Research Department of Applied Physiology, Mossakowski Medical Research Institute Polish Academy of Sciences, Warsaw, Poland

**Keywords:** antioxidant defense, brain, developmental nanotoxicity, kidney, liver, oxidative stress, polystyrene nanoparticles

## Abstract

**Introduction:**

Global plastic production continues to increase, resulting in widespread environmental contamination with plastic waste that gradually breaks down into small particles. Micro- and nanoplastics (MPs/NPs) have recently attracted considerable attention. However, the mechanisms of MPs/NPs toxicity, particularly regarding their developmental impact, remain largely unexplored.

**Methods:**

Two-week-old rats were administered a low dose (1 mg/kg b.w.) of small fluorochrome-labelled polystyrene nanoparticles (PS-NPs) (25 nm) by oral gavage for 3 weeks.

**Results:**

Findings demonstrate that PS-NPs accumulated within the liver, kidneys, and brain, with the highest fluorescence intensity found in the liver and lowest in the brain, as confirmed by the IVIS system. Ultrastructural analysis showed tissue-specific alterations to mitochondrial dynamics, including an excess of small forms in the liver and kidney, and elongated forms in the brain, accompanied by mitochondrial swelling and membrane damage. Markers of oxidative stress, such as significantly increased protein oxidation, were found in all organs, while in the liver, an additionally increased level of lipid peroxidation product (MDA) was observed. Results further showed tissue-specific variations in enzymatic antioxidant systems. While all enzymes examined were upregulated in the kidney, highlighting an active defense, the liver exhibited a notable decrease in antioxidant defense, making it the most vulnerable organ to PS-NPs exposure. In the brain, antioxidant mechanisms were less effective compared to the kidney, despite the lowest PS-NPs deposition rate.

**Discussion:**

While activation of the cellular transcriptional machinery was observed, it was not reflected in protein overexpression, indicating that the immature brain also seems to be susceptible to the adverse effects of PS-NPs.

## 1 Introduction

Global plastic production has increased dramatically in recent decades and its use is expected to continue to grow. Plastics are versatile and essential in many industries. Due to their low production costs and attractive physicochemical properties, plastics have also become ubiquitous in everyday products. With the increasing use of plastics, the world is facing the problem of environmental pollution. It is predicted that by 2050, approximately 12 billion tons of plastic will be released into the environment (Suaria et al., 2016). Poor degradation of plastic waste and limited recycling have led to its significant accumulation in aquatic and terrestrial environments where it decomposes to micro- (MPs; <5 mm) and nanoparticles (NPs; <0.1 µm) under the influence of processes such as weathering, ultraviolet radiation, mechanical abrasion, and biodegradation, contributing significantly to environmental pollution (Kokalj et al., 2021). It is estimated that between 4.8 and 12.7 million metric tons of plastic waste enters the oceans per year (Jambeck et al., 2015) and is detected in other ecosystems, including the atmosphere (Amato-Lourenço et al., 2022), soil (Chen et al., 2022), rivers (Yuan et al., 2022) and even food (Yuan et al., 2022), which is susceptible to MPs/NPs contamination during processing and packaging (Lin et al., 2022). Common MPs/NPs in food and the environment include polyethene-terephthalate (PET), polyethene (PE), polypropylene (PP), and polystyrene (PS) (Ali et al., 2024; Karbalaei et al., 2018). PS is a widely used thermoplastic material, commonly found in disposable cups, packaging, electronics, and personal care products due to its low cost, ease of production, and potential for chemical modification (Song et al., 2020; Sun et al., 2021; Xu et al., 2021). It is one of the most frequently produced polymers that contributes significantly to environmental pollution (Rubio et al., 2020). Studies have shown that humans may ingest 39,000 to 52,000 plastic particles per year (Cox et al., 2019). These amounts appear to be underestimated, as recent studies indicate that the total burden of human exposure to MPs/NPs may be as high as 2.93 × 10^10^ particle/year (Domenech and Marcos, 2021). The estimated daily and weekly intake is 9 mg (Kik et al., 2020) and 0.1–5 g (Senathirajah et al., 2021) per person, respectively.

The ubiquity of MPs/NPs and their persistence in the environment have attracted considerable attention due to increased human exposure and potential long-term health risk. Growing evidence indicates that, due to their small size and high reactivity, MPs/NPs may accumulate in organisms after ingestion (Barría et al., 2020; Du et al., 2024; Gigault et al., 2018), potentially exerting adverse effects on mammalian homeostasis (Covello et al., 2024).

Several studies have demonstrated that polystyrene nanoparticles (PS-NPs) can penetrate biological barriers, including the intestinal barrier and the blood-brain barrier (BBB), depending on NPs characteristics such as size, charge, and surface chemistry, and become retained in various organs (Kulkarni and Feng, 2013; Walczak et al., 2015b). This observation raises questions about how specific tissues respond to the presence of MPs/NPs once they have accumulated.

Studies conducted on *in vitro* and *in vivo* models using different organisms (Ahmed et al., 2022; Azarm-Karnagh et al., 2025; Visalli et al., 2021; Zhou et al., 2023) have shown the presence of biomarkers related to oxidative stress in cells/organs exposed to PS-NPs, as well as various profiles of changes in antioxidant defense mechanisms. The picture emerging from currently available data indicates that PS-NPs cause oxidative stress, but its course depends on many factors related to the properties of the nanoparticles themselves as well as the organisms tested. Therefore, the mechanisms of NPs toxicity require further investigation. In particular, the developmental toxicity of MPs/NPs has not yet been sufficiently characterized, which rises concerns in light of the knowledge that immature organisms are much more susceptible to the toxic effects of various nanoparticles than adult organisms. Age is a factor that strongly determines the absorption, metabolism, distribution, and target organs of harmful substances, including NPs, due to the lower functionality of organs and tissues (Strużyńska et al., 2022). The impact of MPs/NPs on the developing brain is of particular concern due to the intense cell proliferation and synaptogenesis occurring during brain maturation and the immaturity of antioxidant mechanisms.

Therefore, in this study, we analyze the biodistribution of small (25 nm) PS-NPs in immature rats subjected to 3-week-exposure and elucidate in detail one of the potential mechanisms underlying the toxic effects of nanoplastics, which is the induction of oxidative stress in different organs of exposed animals. Using prolonged daily exposure to a low dose (1 mg/kg b.w./day) of PS-NPs via the oral route, we model the environmental conditions relatively accurately (Senathirajah et al., 2021), providing fundamental knowledge on human health risks, with particular emphasis on the developmental period. By focusing on immature organisms, we aim to fill the existing gap in understanding the mechanisms of PS-NPs toxicity during development.

## 2 Materials and methods

### 2.1 Characteristic of PS-NPs

Two types of PS-NPs (25 nm) (Cat. No. PST25A; FRP25A) were purchased from Lab261 (Palo Alto, CA). A batch of PS-NPs labelled with a red fluorophore with fluorescence spectra 545/566 nm was specifically used to assess the accumulation and distribution of NPs in rats, while a batch without fluorescent labeling was used to assess oxidative stress.

According to the manufacturer, the red fluorophore-labelled PS-NPs contain internally encapsulated dye, within the hydrophobic core of the polystyrene matrix, that provides stable fluorescence resistant to photobleaching (website: https://www.lab261.com/pages/fluorescent-polystyrene-microspheres).

The shape and size of PS-NPs were confirmed by transmission electron microscopy (TEM) (JEM-1200EX, Jeol, Japan) equipped with an EDS INCA analyzer as previously described (Adamiak et al., 2024). The dispersion was confirmed by measuring the zeta potential and hydrodynamic diameter of PS-NPs in various pH and temperatures, including physiological conditions, using a dynamic light scattering (DLS) instrument as previously described (Adamiak et al., 2025).

### 2.2 Animals and experimental design

An experimental model of developmental exposure was developed, in which immature rats were subjected to an environmentally plausible dose of PS-NPs calculated based on available literature data (Du et al., 2024; Leslie et al., 2022; Priya et al., 2022; Senathirajah et al., 2021), which is discussed further in subsection 4.1.

Three litters of two-week-old Wistar pups (total n = 36) with their mothers were obtained from the Animal Breeding Center of the Mossakowski Medical Research Institute Polish Academy of Sciences (Warsaw, Poland). Animals were housed in ventilated, temperature-controlled cages under standardized sterile conditions with a 12-h light/dark cycle and unlimited access to food and water.

At postnatal day 14 (PND 14), both male and female pups were randomly allocated into control and experimental groups to minimize litter-specific effects. This “Split-Litter Design” was chosen as a recommended randomization strategy, in which each litter serves as its own “control”. Genetic traits and maternal care affect both groups (control and experimental) equally. The control group consisted of 15 rats, including 8 females and 7 males. The test group (PS-NPs) comprised 21 rats, including 11 females and 10 males.

Animals were administered 0.9% NaCl and 1 mg/kg b.w. PS-NPs, respectively. The appropriate solution was given daily by oral gavage for 21 days. During exposure, pups were returned to their mothers after receiving the solutions and remained with them until they were able to eat on their own. They were then separated and placed in cages of two. The administered dose of PS-NPs was adjusted each day depending on the body weight gain of the rats. Daily observations included food consumption, body weight and animal welfare. On PND 35, rats were sacrificed by decapitation, and selected organs (liver, kidney and brain) were sectioned, frozen in liquid nitrogen, and stored at −80 °C. In addition, eight animals were anesthetized and perfused for TEM analysis. In the case of the brain, the cerebral cortex and hippocampus were collected for ultrastructural studies. The whole brain (excluding olfactory bulbs and brainstem) was harvested and frozen in liquid nitrogen for further biochemical and molecular analyses.

All experimental procedures using animals were carried out in accordance with the EU Directive for the Care and Use of Laboratory Animals (Directive 2010/63/EU) and were approved by the II Local Experimental Animal Care and Use Committee in Warsaw (permit no WAW2/043/2022).

In addition, to adhere to the 3Rs principles of animal welfare (specifically Reduction) and minimize the total number of animals sacrificed, the organs (liver, kidney, and brain) analyzed in the present study were partially collected from the same animal cohort described in our previous study on intestinal homeostasis (Augustyniak et al., 2025). No data or organ endpoints overlap between the two manuscripts.

### 2.3 Analysis of PS-NP-derived fluorescence in organs

To confirm the presence of PS-NPs in tissues isolated from exposed rats, the fluorescence images were acquired using the IVIS® Lumina III Series Imaging System (Revvity, MA). To separate the specific fluorescence signal of PS-NPs from tissue autofluorescence, the spectral unmixing analysis was performed using the Living Image 4.7.4 software. All images were acquired in field of view, and the height value was set at 1.5 cm. Regions of interest (ROIs) were manually marked along the edges of the imaged organs. Finally, Average Radiant Efficiency (ARE) was measured [photons/second/cm^2^/steradian]/[µWatt/cm^2^].

### 2.4 Confocal imaging of PS-NP-derived fluorescence

The frozen tissue samples from the control and exposed groups were cut into 20 μm-thick sections on a cryostat (Leica CM1860 UV, Leica Biosystems, Nussloch, Germany), placed on slides (Thermo Fisher Scientificc, Waltham, MA, USA), rinsed in PBS and fixed in 4% paraformaldehyde for 20 min. In the case of the brain, coronal sections encompassing the cerebral cortex and the dorsal hippocampus were used to visualize the presence and cellular distribution of PS-NPs. Samples were then incubated in 10% goat serum in PBS containing 0.25% Triton X-100% and 0.1% bovine serum albumin for 60 min, after washing with PBS. In addition, cell nuclei were stained with Hoechst 33258 (Sigma‒Aldrich, St. Louis, MO, USA; 1 μg/mL). To visualize the presence and distribution of PS-NPs in tissues, PS-NP-derived immunofluorescence analysis was performed in six sections taken from each of three animals per group. Immunofluorescence intensity was measured in similar regions of interest (ROIs) to compare the control and PS-NP-exposed groups. Additional staining with cell-specific markers was performed in brain samples to visualize the cellular distribution of PS-NPs. Anti-MAP2 (Cat. No. M4403; Sigma-Aldrich, 1:750) and anti-GFAP (Cat. No. G3893 Sigma-Aldrich, 1:500) primary antibodies were used to label neurons and astrocytes, respectively. A secondary antibody, goat anti-Mouse IgG (H + L) Superclonal™ conjugated with Alexa Fluor™ 488 (Cat. No. A28175 Invitrogen, 1:500), was then used. Images were taken using a LSM780/Elyra PS.1 confocal laser scanning microscope with ZEN 2.6 software (Carl Zeiss, Jena, Germany) at the Laboratory of Advanced Microscopy Techniques, MMRI PAS.

### 2.5 TEM analysis

The animals were anaesthetized with nembutal (80 mg/kg b.w.) and perfused transcardially, first with 0.9% NaCl in 0.01 M sodium–potassium phosphate buffer (pH 7.4), and then with 2% paraformaldehyde and 2.5% glutaraldehyde in 0.1 M cacodylate buffer (pH 7.4). Tissue samples (brain, liver and kidney) were subjected to routine tissue processing for TEM, i.e., they were post-fixed in 1% OsO4 solution, dehydrated in ethanol and rinsed in acetone. After dehydration and embedding in epoxy resin (Epon 812), ultra-thin sections were prepared using a Leica EM UC735 ultramicrotome and then stained with 9% uranyl acetate and lead nitrate. Micrographs were taken using a transmission electron microscope (TEM) (JEM-1200EX, Jeol, Tokyo, Japan) equipped with a MORADA digital camera and iTEM 1233 software (Olympus Soft Imaging Solutions, GmbH, Münster, Germany).

### 2.6 Lipid peroxidation assay

The levels of lipid peroxidation in tissue samples were determined using an MDA assay kit (Merck; Cat. No. MAK085). Approximately 10 mg of tissue was homogenized in MDA Lysis Buffer containing butylated hydroxytoluene (BHT). The homogenized samples were then centrifuged at 13,000 × g for 10 min to remove insoluble debris. After centrifugation, 200 µL of supernatant was transferred into microcentrifuge tubes. To form the MDA-thiobarbituric acid (TBA) adduct, 600 µL of TBA solution was added to each sample and standard vial. The reaction mixture was incubated at 95 °C for 60 min to ensure proper adduct formation. After incubation, samples were cooled to room temperature in an ice bath for 10 min. For analysis, 200 µL of each reaction mixture was transferred into a 96-well plate and absorbance was measured at 532 nm. Background values were subtracted from all readings. The slope of the standard curve was used to calculate the MDA concentration. Results are expressed as a percentage of control values.

$$\text{Concentration of MDA}=\frac{Amount\;of\;MDA\;in\;sample\;\left(nmol\right)}{Sample\;volume\;addded\;into\;the\;wells\;\left(mL\right)}\times Dilution\;Factor\;\left[nmol/mL\right]$$

### 2.7 Protein carbonyl group content

Tissues were homogenized manually using a conical homogenizer in distilled water. The samples were then centrifuged at 13,000 × g for 5 min at 4 °C. To remove interfering nucleic acids, the supernatant was treated with a 10% streptozocin solution, incubated for 15 min at room temperature, and centrifuged again at 13,000 × g for 5 min at 4 °C. The supernatant was used for analysis according to the manufacturer’s instruction (Protein Carbonyl Content Assay Kit; Cat. No. MAK094; Merck). DNPH Solution was added to the samples and incubated for 10 min at RT. Next, 87% TCA Solution was added and incubated on ice for 5 min. Samples were centrifuged at 13,000 × g for 2 min, and the supernatant was discarded. Cold acetone was added to the pellet, and the samples were then sonicated and incubated for 5 min at −20 °C. The samples were centrifuged at 13,000 × g for 2 min, and the supernatant was discarded again. The washing step with acetone was performed twice. Finally, a 6M Guanidine Solution was added to the samples and briefly sonicated. The absorbance was measured at 375 nm and compared to the protein standard curve (BCA) described above. Absorbance values were calculated based on the formulas below and expressed as % of control values.

$$\text{Carbonyl content per well}=\frac{Absorbance\;at\;375\;nm}{6.364}\times 100\;\left[nmol/well\right]$$

$$\text{Carbonyl }content=\frac{Amount\;of\;carbonyl\;in\;sample\;well}{Amount\;of\;protein\;from\;standard\;well\times 20}\times 1000\times \text{Dilution Factor }\left[nmol/mgprotein\right]$$

### 2.8 DNA damage - 8-OHdG assay

Before analysis, DNA was isolated from each tissue homogenate using the GeneJET™ Genomic DNA Purification Kit (Cat. No. K0722; Thermo Scientific™). Tissues were suspended in Proteinase K digestion solution and incubated for 4h at 56 °C on a rocking platform. After incubation, samples were centrifuged at 12,000 × g for 3 min and mixed with RNase A Solution for 10 min at RT. Lysis buffer and 50% ethanol were added, and the mixture was transferred to the column, which was centrifuged at 6,000 × g for 1 min. The columns were then washed twice (Wash Buffer I and II), and centrifuged first at 8,000 × g for 1 min, and then at 13,000 × g for 3 min. The final step was performed to elute genomic DNA from the column into an Eppendorf tube by adding Elution Buffer and centrifuging at 8,000 × g for 1 min. Subsequently, dsDNA quantification (ng/µL) was performed on a DeNovix DS-11 FX spectrophotometer and adjusted for analysis.

The isolated genomic DNA was used for measurement using the DNA Damage Competitive ELISA Kit (Cat. No. EIADNAD; Thermo Scientific™). Samples were pipetted into strips. The assay included an NSB (Non-Specific Binding) well with assay buffer only. 8-Hydroxy-2′-deoxyguanosine Conjugate was added to each well before the addition of 8-Hydroxy-2′-deoxyguanosine antibody (except for the NSB wells). The plate was incubated for 2h with shaking at RT. After incubation, the plate was washed 4 times using Wash Buffer. TMB Substrate was added to each well with further incubation for 30 min at RT. Finally, Stop Solution was added, and the absorbance was measured at 450 nm. Concentrations of 8-Hydroxy-2′-deoxyguanosine (pg/mL) are presented as % of control samples.

### 2.9 GSH and total glutathione (GSH + GSSG) assay

The Glutathione GSH/GSSG Assay Kit (Cat. No. MAK440; Merck) was used to accurately measure the total peptide amount. The analysis was performed after homogenization of rat tissues in 50 mM Phosphate buffer (pH = 7.0) containing 1 mM EDTA. Samples were centrifuged at 10,000 × g for 15 min at 4 °C. Before the reaction, the samples were deproteinized by mixing with 5% meta-Phosphoric Acid Solution (MPA). The mixture was centrifuged at 14,000 × g for 5 min at 4 °C. The supernatant was mixed with Assay Buffer and transferred to a 96-well plate with Working Reagent. Total glutathione concentration (µM) was determined by measuring the change in absorbance values between two time points (0 and 10 min) at 412 nm and comparing it with a glutathione standard curve. Final values are shown as % of control.

Reduced glutathione (GSH) was measured by a direct procedure based on the Glutathione Assay Kit (Cat. No. MAK517; Merck). Tissues were homogenized in ice-cold phosphate buffer containing 1 mM EDTA. Samples were centrifuged at 10,000 × g for 15 min at 4 °C to obtain the supernatant. The samples were then mixed with Reagent A for color reaction and deproteination (ratio 1:1), and next with Reagent B (5,5′-dithiobis(2-nitrobenzoic acid)). After 25 min of incubation, the absorbance was measured at 412 nm. GSH concentration (µM) was calculated based on the calibrator’s value and presented as % of the control.

### 2.10 Protein measurement

The protein concentration in each sample was determined using Pierce™ BCA Protein Assay Kit (Cat. No. 23225; Thermo Scientific™). In each reaction working reagent (WR) prepared in a ratio of 50:1 (Reagent A:B) was used. The samples were then pipette into microplate wells with WR and mixed on a plate shaker for 30 s. The plate was incubated at 37 °C for 30 min, and the absorbance was measured at 562 nm on a plate reader (FLUOstar Omega; BMG LABTECH) with a standard curve based on the BSA concentration.

### 2.11 Catalase activity measurement

Tissue was homogenized in Assay Buffer and centrifuged at 10,000 × g for 15 min at 4 °C. The sample supernatant was used for analysis using the Catalase Colorimetric Activity Kit (Cat. No. EIACATC; Invitrogen™). First, Hydrogen Peroxide Reagent was added to the well and incubated for 30 min at RT. After incubation, the same volume of Substrate and HRP was added. The mixture was then incubated for 15 min at RT and the absorbance was measured at 560 nm. The catalase activity (U/mL) was calculated from the standard curve and presented as % of control.

### 2.12 SOD activity measurement

Superoxide dismutases (SODs) are a family of metal ion-containing enzymes. Their activity was measured using the Superoxide Dismutase Activity Assay Kit (Cat. No. CS0009; Merck). Before assay, tissues were homogenized in ice-cold buffer (0.1 M Trizma®-HCl, pH 7.4, containing 0.5% Triton™X-100, 5 mM mercaptoethanol, and protease inhibitors). After centrifugation at 14,000 × g for 5 min at 4 °C, the supernatant was applied and mixed with WST Working Solution and Xanthine Oxidase Working Solution. Samples were incubated for 30 min at RT, and absorbance was measured at 450 nm. Based on the OD values of the treated and standard samples, the SOD inhibition rate was calculated after background subtraction, using the equation below, and was expressed as a % of control.

$$\text{SOD inhibition rate}=\frac{Absorbance\;of\;No\;SOD\;control-Absorbance\;of\;Sample}{Absorbance\;of\;No\;SOD\;control}$$

### 2.13 GSH-Px activity measurement

Rat tissues were homogenized in PBS. Centrifugation was performed at 10,000 × g for 10 min at 4 °C. The supernatant was transferred, and enzyme activity was determined by measuring the consumption of reduced glutathione with the Glutathione Peroxidase (GSH-Px) Activity Kit (Cat. No. EEA010; Invitrogen™). The reaction was carried out under two conditions: non-enzymatic (only 1 mmol/L GSH Standard) and enzymatic (1 mmol/L GSH Standard with tissue sample). Both samples were heated in a water bath at 37 °C for 5 min. After incubation, the Stock Application Solution was added to each of them, and they were heated again under the same conditions. Samples were centrifuged at 3,100 × g for 10 min. Each supernatant was transferred to a 96-well plate, and Phosphate and DTNB Solution was added to each well. After 5 min of incubation, the absorbance at 412 nm was measured, and the GSH-Px activity (U/mg protein) was calculated based on the equation presented below. The final results are presented as % of control.

$$\text{GSH}-\text{Px activity}=\left(\Delta \;Absorbance\;\left[Nonenzymatic-Enzymatic\right]-The\;intercept\;of\;standard\;curve\right)÷The\;slope\;of\;standard\;curve\times \frac{0.23+Sample\;volume\;in\;reaction}{0.03+Sample\;volume\;in\;reaction}÷\left(Sample\;volume\;in\;reaction\times Concentration\;of\;protein\;in\;sample\right)\times Dilution\;Factor$$

### 2.14 RT-PCR analysis

Total RNA was extracted from each tissue using TRIzol™ Reagent (Cat. No. 15596018; Invitrogen™). Chloroform was added to the samples, and the mixture was centrifuged at 12,000 RPM (Thermo Scientific Heraeus Megafuge 16R Centrifuge) for 10 min at 4 °C. The aqueous phase was separated, mixed with propanol, and incubated at −20 °C. After incubation, samples were centrifuged at 12,000 RPM for 10 min at 4 °C. Subsequently, the samples were washed 3 times with 75% ethanol. Finally, the pellet was diluted in DEPC water, and the RNA concentration (ng/µL) was measured on a DeNovix DS-11 FX spectrophotometer. RNA (2 µg) was reverse transcribed using random primers and the High-Capacity cDNA Reverse Transcription Kit (Cat. No. 4368814; Applied Biosystems™). RT-PCR conditions included the following steps: incubation at 25 °C (10 min), at 37 °C (120 min), and at 85 °C (5 min). Rat-specific primers for: *Cat* (Rn01512560_m1), *Sod-1* (Rn00566938_m1), *Sod-2* (Rn00690588_g1), and *Gsh-Px4* (Rn00820818_g1) from Applied Biosystems™ were used. Quantitative real-time PCR (qPCR) analysis was conducted using TaqMan™ Gene Expression Master Mix (Cat. No. 4369016; Applied Biosystems™). TaqMan PCR Master Mix, TaqMan probe and 5 µL of RT product were mixed in a total volume of 20 µL. The qPCR cycle conditions were as follows: initial denaturation at 95 °C for 10 min, 45 cycles of 95 °C for 15 s, and 60 °C for 1 min. Relative expression of enzyme mRNAs was normalized to rat *ACTB* (actin) (Cat. No. 4352340E; Applied Biosystems™) as a reference gene and calculated based on the ΔΔCt method.

### 2.15 WB analysis

Tissues were homogenized in RIPA buffer (Cat. No. 89901; Thermo Scientific™) with protease (Cat. No. P8340; Merck) and phosphatase (Cat. No. P0044; Merck) inhibitors. The samples were then centrifuged at 12,500 RPM for 20 min at 4 °C (Thermo Scientific Heraeus Megafuge 16R Centrifuge). The supernatant of each sample was mixed 1:1 with Laemmli buffer, followed by protein denaturation.

Sodium dodecyl sulfate-polyacrylamide gel electrophoresis was performed. Samples containing 30 µg of protein were prepared in loading buffer, separated on 10% (CAT) and 15% (SOD1, SOD2 and GPX4) polyacrylamide gel, and then transferred to a nitrocellulose membrane (Cat. No. 28906835; Amersham Hyperfilm ECL). Proteins were fixed on the membrane in TBS buffer containing 0.1% Tween-20% and 5% non-fat milk (TTBS) for 1.5 h. After washing in TTBS buffer (3 × 10 min), the blots were first incubated with primary antibodies against: CAT (Cat. No. PA5-29183; rabbit; 1:1000; Invitrogen); SOD-1 (Cat. No. PA5-23245; rabbit; 1:10,000; Invitrogen); SOD-2 (Cat. No. PA5-30604; rabbit; 1:500; Invitrogen), and GSH-Px4 (Cat. No. MA5-32827; rabbit; 1:10,000; Invitrogen) for 2 h, followed by HRP-conjugated anti-rabbit secondary antibody (1:10,000; Merck). Beta-actin (Cat. No. ab49900; Abcam) was used to confirm equivalent loading of each sample. The bands were visualized using a chemiluminescence ECL kit (Westar Hypernova; Cat. No. XLS149) and exposed in Fusion Fx (Vilber Lourmat). Densitometric analysis was performed using ImageJ software.

### 2.16 Statistical analysis

Statistical analyses and graph preparation were performed using GraphPad Prism software (version 11). Data are expressed as means ± standard deviation (SD). The normality of data distribution was verified using the Shapiro-Wilk test to determine whether parametric or non-parametric tests were appropriate. For individual binary comparisons, the Student’s t-test was used for normally distributed data, while the Mann-Whitney test was applied for non-parametric data sets. The level of statistical significance was set at p < 0.05 for all analyses.

Three litters, each consisting of approximately 12 pups, was used in the study. Importantly, the pups in both the control and PS-NP-treated groups were mixed, meaning they originated from different litters. An important aspect is the fact that our use of animals is limited due to the strict requirements set by the local ethics committee. For this reason, we used “Split-Litter Design” as the recommended randomization strategy, in which each litter served as its own control, i.e., both male and female pups were randomly allocated into the control and experimental groups to minimize litter-specific effects. In addition, the number of pups used in each analysis was given in the legends of individual figures.

## 3 Results

### 3.1 Characteristics of PS-NPs

Commercially available 25 nm PS-NPs conjugated with a red fluorochrome with an Ex/Em spectrum of 545/566 nm were used in this study. Our previous analyses, conducted to confirm the manufacturer’s information on the size, shape and the dispersion of PS-NPs, revealed that the purchased batch contained spherical particles with an average diameter of 24 nm and low zeta potential, indicating that they did not aggregate in the original solution and in the solution imitating physiological conditions (pH = 7.4; 37 °C) (Adamiak et al., 2025; 2024).

### 3.2 Biodistribution of nanoplastics in tissues of exposed animals

Animals were weighed daily to adjust the PS-NP dose proportionally to body weight. After 3-week-exposure, there were no statistically significant differences in body weight between the control and experimental groups, which is consistent with previous reports (Du et al., 2024; Rafiee et al., 2018).

*Ex vivo* imaging of fluorescently labelled PS-NPs in isolated organs was performed using the IVIS® Spectrum system. This advanced preclinical optical imaging system enables imaging and semi-quantification of commonly used fluorophores. Thanks to the application of advanced spectral unmixing algorithms, the effect of tissue autofluorescence is significantly reduced.

After prolonged 3-week oral exposure to red fluorochrome-labelled PS-NPs, fluorescence mapping was performed in organs such as brain, liver and kidney isolated from experimental animals. The pseudo-color images of organs obtained from the control group showed no significant presence of red fluorescence.

In organs isolated from exposed animals, the color gradually increased from red to yellow. The most intense yellow signal was observed in the liver, while weaker (yellow-red) signals were detected in the kidney and brain (Figures 1A–C). Measurements and statistical analysis of fluorescence signals from regions of interest (ROIs) revealed differences between control and exposed animals (Figures 1a–c). Significantly increased fluorescence intensity compared to controls was observed in all organs tested, i.e., in brain (p = 0.0187 vs. control), kidney (p = 0.0007) and liver (p = 0.0092). Significant differences in signal intensity (expressed as Average Radiant Efficiency [p/sec/cm^2^/sr]/[µW/cm^2^]; ARE) between organs were also observed in the exposed groups. The order of magnitude of ARE value was in the range of 10^5^–10^7^ and increased in individual tissues as follows: ∼7.5 × 10^5^ (brain), ∼3.7 × 10^6^ (kidney), ∼1 × 10^7^ (liver), which indicates the highest accumulation of PS-NPs in the liver and the lowest in the brain. ARE values reflect a semi-quantitative rank order of relative tissue retention (liver > kidney > brain).

**FIGURE 1:**
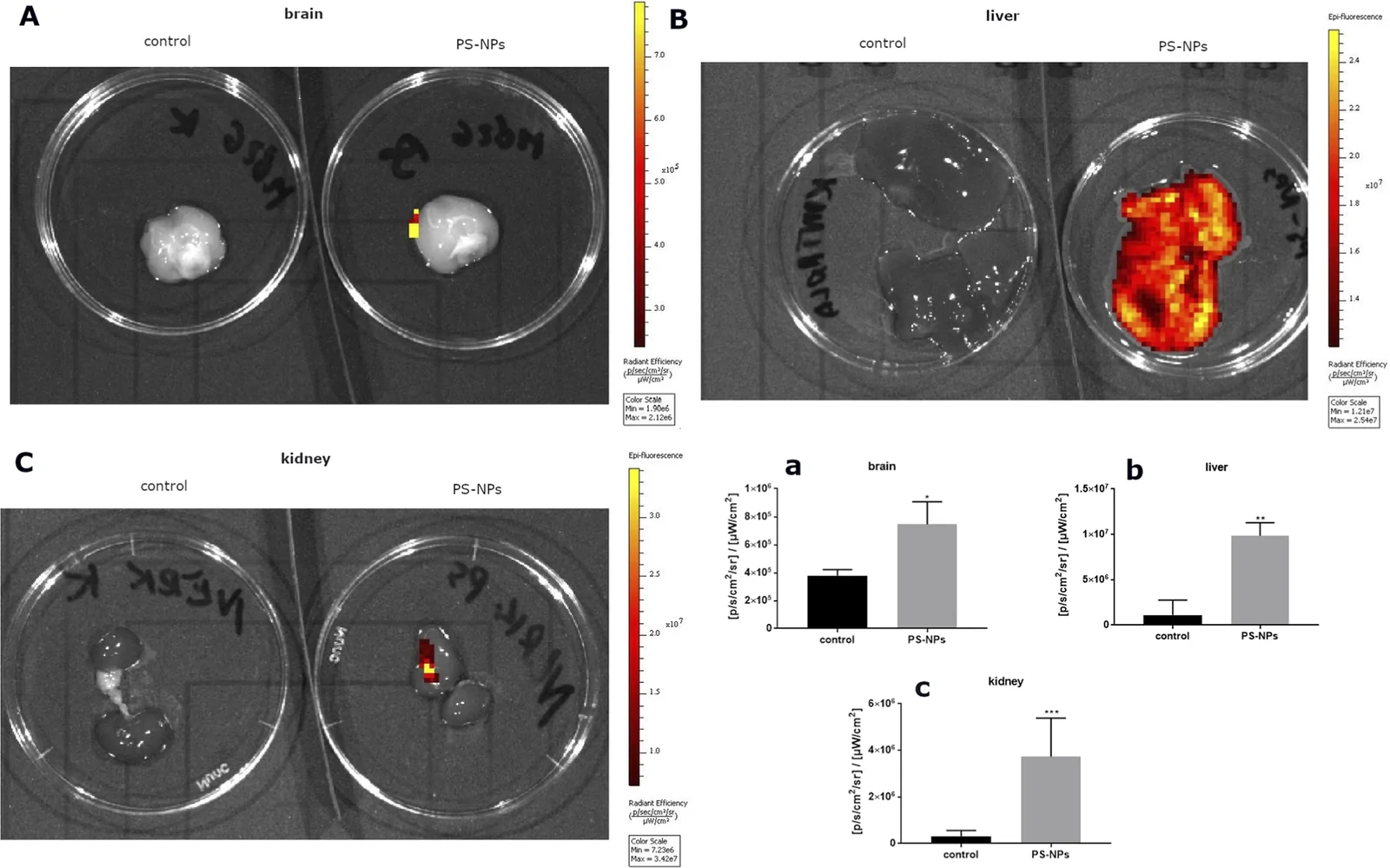
Accumulation of PS-NPs in organs based on *ex vivo* imaging of fluorescence intensity using IVIS system. Representative spectrally unmixed images of fluorescently labelled PS-NPs in brain **(A)**; liver **(B)**; kidney **(C)**. Each image represents the corresponding organ isolated from control (left panel) and PS-NP-exposed (right panel) animals. The pseudo-color scale is expressed as a gradient of Average Radiant Efficiency (ARE) [p/sec/cm^2^/sr]/[µW/cm^2^]. The graphs represent the mean ± SD of fluorescence intensity measured in organs isolated from three different control and PS-NP-exposed animals **(a,b,c)**; *p < 0.05; **p < 0.01; ***p < 0.001 (Unpaired T-test).

Additionally, samples of all examined organs were subjected to an immunohistochemical procedure and analyzed by confocal microscopy. Background red fluorescence was observed in control tissues, which was relatively high in the kidney and the liver, and lowest in the brain, confirming the results of the IVIS analysis. The presence of red fluorescence representing PS-NPs was visible in all tissues collected from exposed animals (Figure 2A). In the coronal cryosections encompassing cerebral cortex and dorsal hippocampus, additional cell-specific labeling was performed, allowing to confirm the intracellular localization of PS-NPs within both GFAP-positive astrocytes and MAP2-positive neurons (Figure 2B). The PS-NP-derived red fluorescence exhibited a distinct punctate, granular pattern predominantly localized in the perinuclear cytoplasm of both cell types.

**FIGURE 2:**
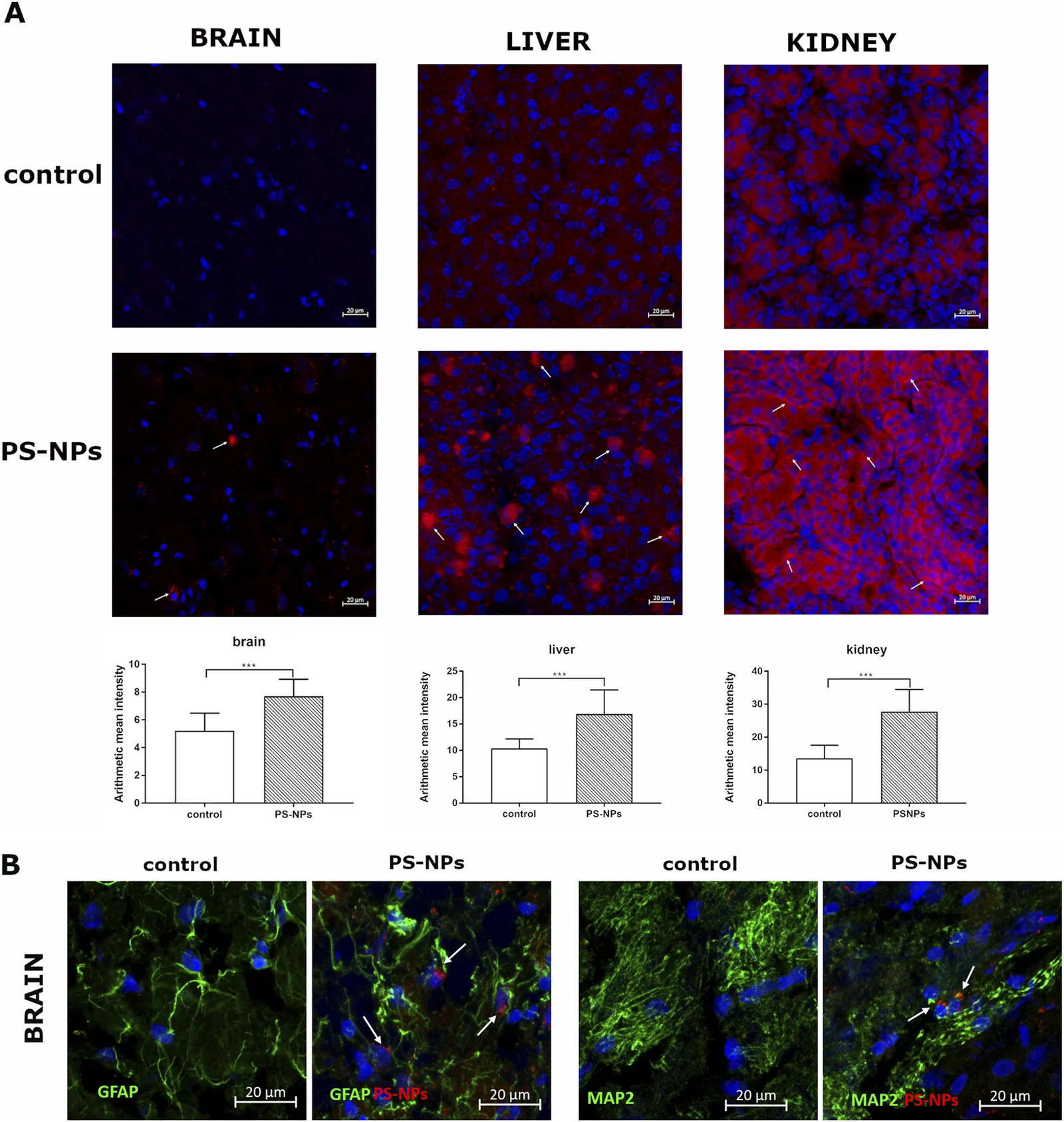
Tissue-specific biodistribution of red fluorochrome-conjugated PS-NPs in the brain, liver and kidney obtained from control and PS-NP-exposed immature rats. **(A)** Representative confocal microscopy images showing the presence of red PS-NP-derived fluorescence in tissues. The graphs represent the mean ± SD of fluorescence intensity measured in the corresponding regions of interest (ROIs) in 6 specimens collected from three animals per group; ***p < 0.001 (Unpaired T-test) **(B)** Cell-specific biodistribution of red fluorochrome-conjugated PS-NPs in the cerebral cortex of immature control and PS-NP-exposed rats. Representative images showing the presence of PS-NPs inside neurons (MAP2; green) and astrocytes (GFAP; green). A distinct, punctate and granular fluorescence is observed, distributed intracellularly, with no diffuse extracellular background. Hoechst was used to visualize the nuclei (blue). Bars indicate 20 µm.

### 3.3 Ultrastructural changes in mitochondria induced by nanoplastics in the examined tissues

Since there is a correlation between mitochondrial status and oxidative stress, we first investigated whether exposure to PS-NPs causes ultrastructural changes in mitochondria in the examined tissues. In TEM studies, we observed evidence of PS-NP-induced disruptions of mitochondrial morphology depending on the tissue type. One of the most important mechanisms of mitochondrial quality is maintaining the dynamics between fusion and fission.

In the brain, a shortening of mitochondrial length, characteristic of their division, was observed, resulting in a large number of small, round, often swollen mitochondria (Figures 3A,B). An important and frequent feature was the presence of abnormally elongated forms of mitochondria in nerve fibers (Figures 3A–D), which may possibly reflect the mechanism of fusion.

**FIGURE 3:**
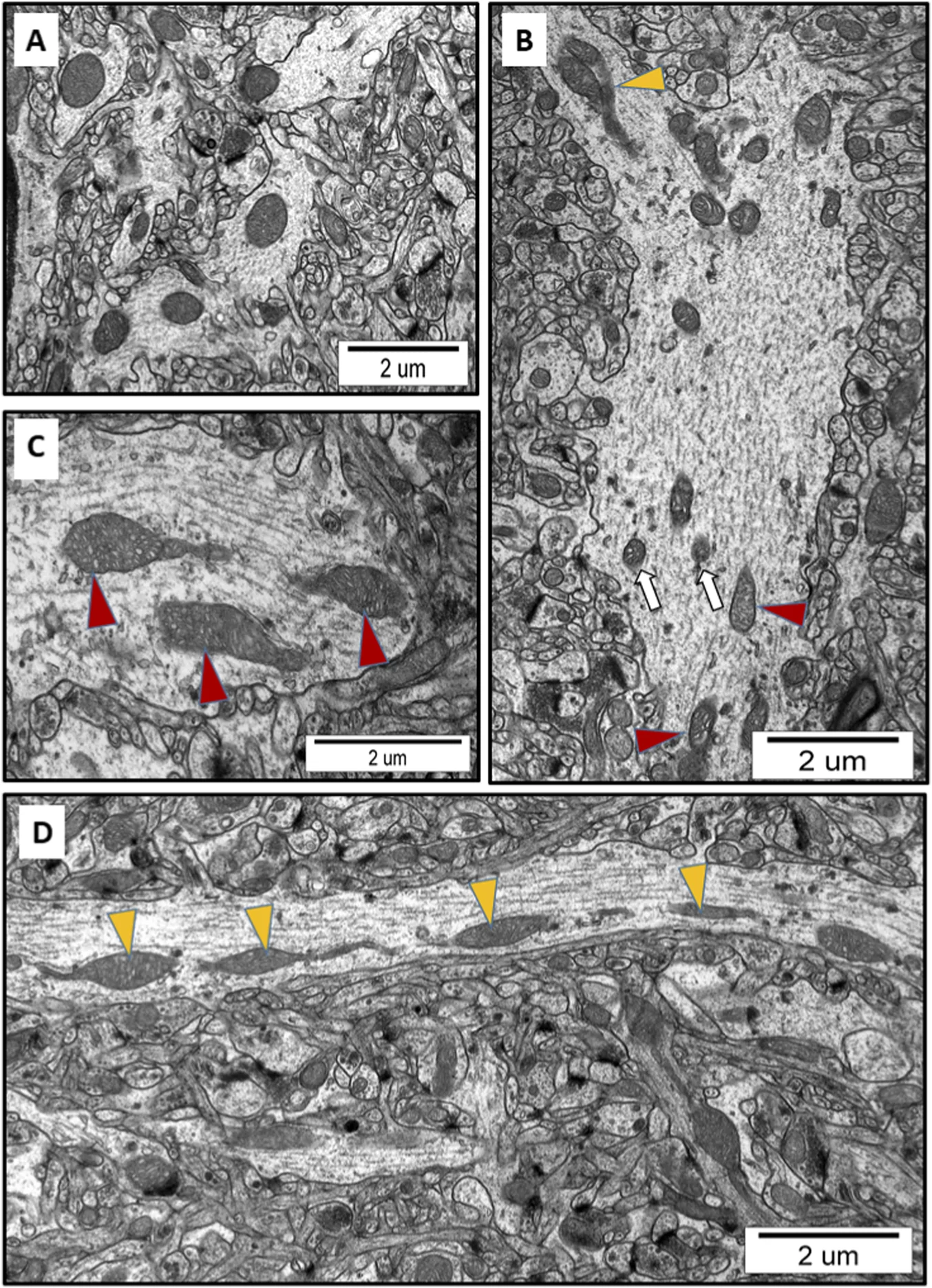
Representative TEM micrographs showing morphological features of mitochondria in control **(A)** and PS-NP-exposed immature rat brain **(B–D)**. Elongated (yellow arrowhead) and small mitochondria (white arrows) in nerve fibers, often swollen (red arrowheads). Images are representative for both cerebral cortex and hippocampus.

Also in the liver, a large number of small, round mitochondria were observed in hepatocytes (Figure 4A). In addition, lucent areas of cytoplasm devoid of glycogen granules, mitochondria and rough endoplasmic reticulum (RER) membranes were also observed in many hepatocytes (Figure 4B). Glycogen granules are a cellular storage form of glucose, found primarily in the liver and muscle tissues, providing a readily available source of energy. Since glycogen synthesis is an energy-intensive process, depletion of its pool in liver cells may suggest energy disorders, which is consistent with the observed changes in mitochondria. Similarly, in some kidney cells, we observed numerous small mitochondria (Figure 5A), some of which were vacuolated and swollen, and had damaged cristae (Figure 5B).

**FIGURE 4:**
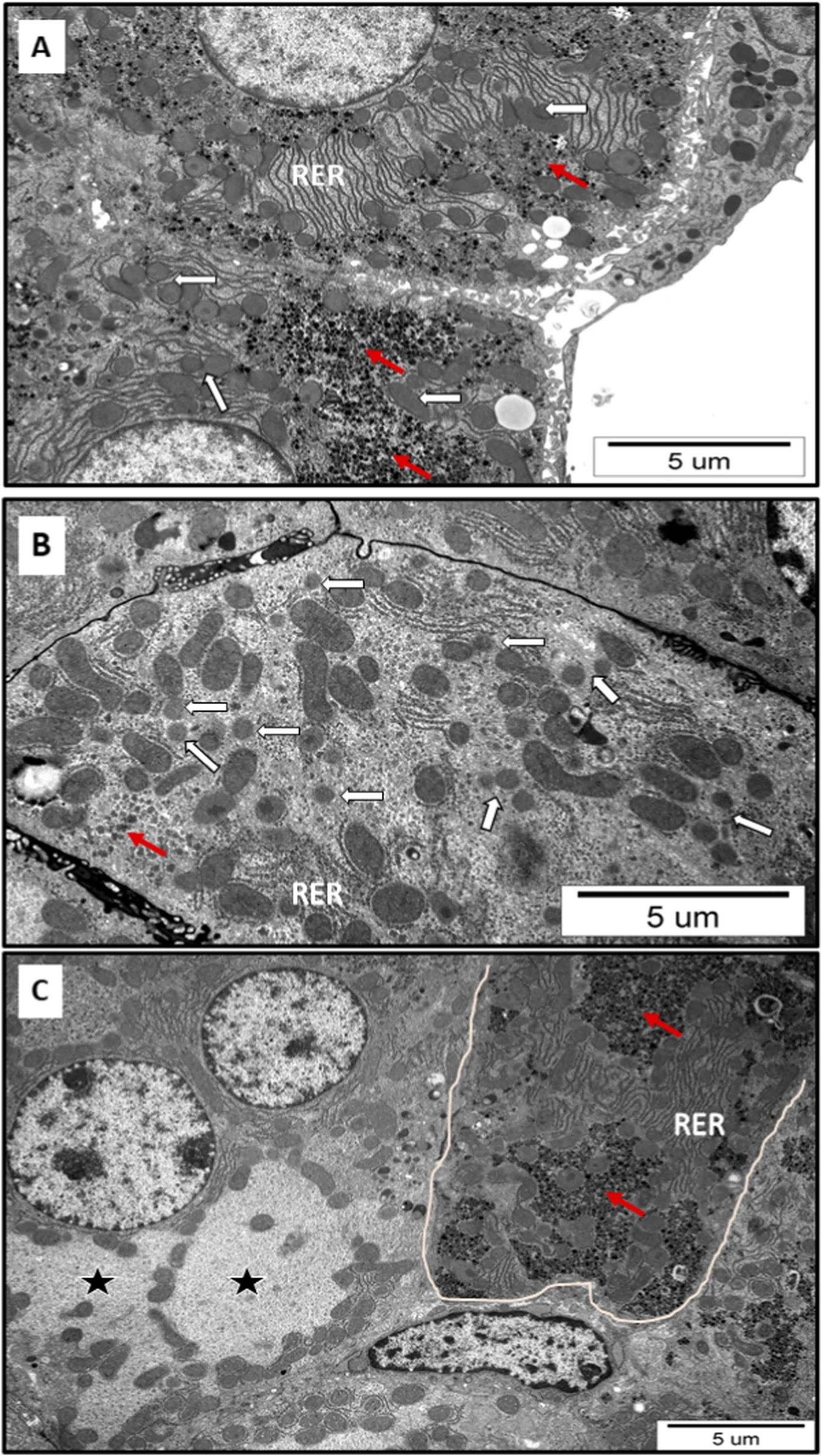
Representative TEM micrographs showing morphological features of liver cells in control **(A)** and PS-NP-exposed immature rats **(B,C)**. **(A)** Normal appearance of hepatocytes - numerous mitochondria of similar size (white arrows) and an appropriate amount of glycogen deposits (red arrows) **(B)** Numerous small mitochondria (white arrows); sparse glycogen granules (red arrow); **(C)** a cell with empty cytoplasmic spaces (asterisks) devoid of glycogen granules and cellular structures such as rough endoplasmic reticulum (RER), lying next to a normally-structured cell (marked with white line) that shows a regular amount of glycogen (dark granules marked with red arrows) and RER.

**FIGURE 5:**
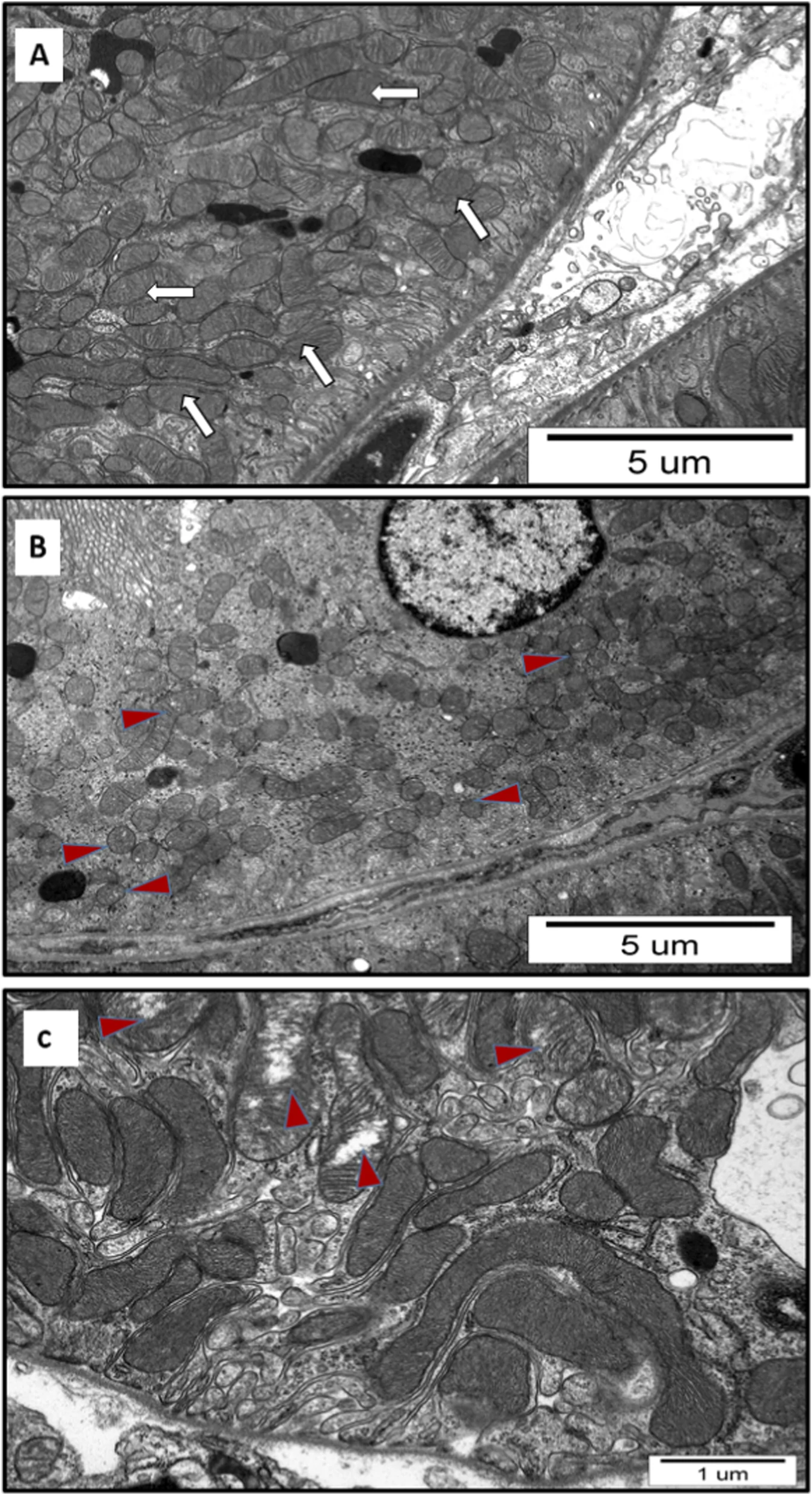
Representative TEM micrographs showing morphological features of kidney mitochondria in control **(A)** and PS-NP-exposed immature rats. **(A)** Normal appearance of cells–numerous mitochondria uniform in size, without signs of edema **(B)** Cell with numerous small mitochondria, frequently swollen (red arrowheads); **(C)** cell with normal-appearing mitochondria, swollen mitochondria with disrupted cristae (red arrowheads) and elongated mitochondrium.

In summary, the results of TEM analysis showed that under conditions of PS-NPs exposure, mitochondrial morphology was disturbed compared to control (Figure 6A) and characterized by vacuolization and changes in mitochondrial biodynamic in all tissues examined (Figures 6B–E), with a PS-NP-induced increase in the number of small mitochondria compared to normal conditions, mainly observed in the liver and kidney, and excessive mitochondrial elongation in the brain tissue. Moreover, in the brain and to a lesser extent in the kidney, mitochondria appear to be dysfunctional, because their cristae often lose normal structure and their outer membranes become damaged (Figures 6E–G).

**FIGURE 6:**
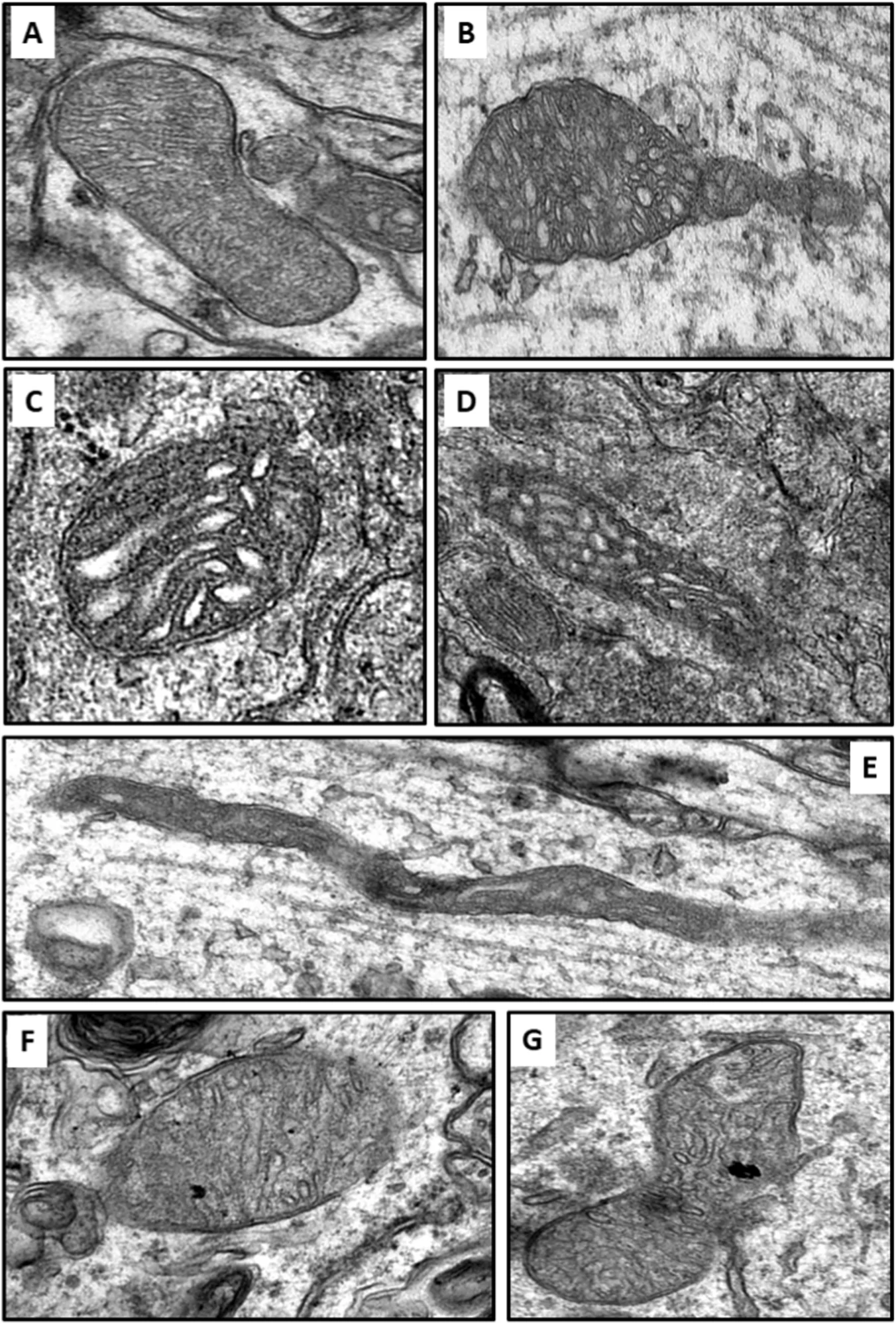
Representative TEM micrographs showing normal mitochondrial morphology in a control rat **(A)** and examples of mitochondrial abnormalities found in the examined tissues of PS-NP-exposed immature rats. Swollen and vacuolated mitochondria with blurred internal structure of cristae **(B–D)**; abnormally elongated mitochondrium, partially swollen **(E)**; fragmented outer mitochondrial membranes and blurred structure of cristae **(F,G)**. The images represent a digitally cropped and enlarged regions of interest (ROI) from the original TEM micrographs to enhance visual clarity of the structural details of mitochondria presented on Figures 3–5 and described in the section Results. Due to the non-uniform digital scaling, the original scale bars are omitted, and the images are intended for qualitative visualization only.

### 3.4 Markers indicative of PS-NP-induced oxidative stress and antioxidant response

We then determined whether PS-NPs could potentially induce oxidative stress in the tissues where they accumulate. Oxidative damage to molecules such as lipids, proteins and DNA was assessed by determining the levels of MDA, carbonyl group content and 8-OHdG, respectively. MDA levels increased significantly compared to the control group exclusively in the liver (p = 0.0165), while remaining unchanged in the brain and kidney (Figures 7A–C).

**FIGURE 7:**
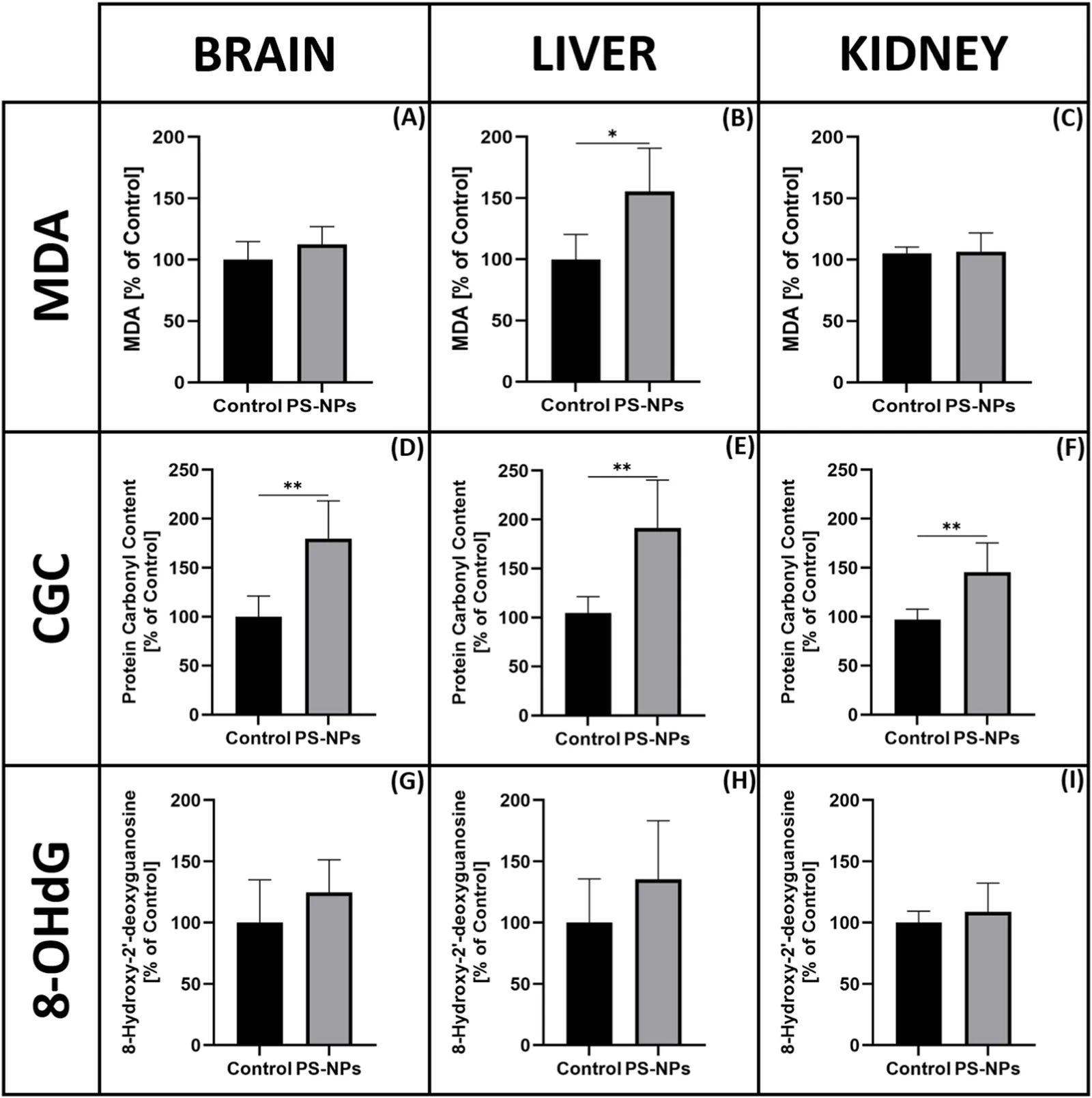
Oxidative stress markers indicating oxidative damage of proteins, lipids and DNA in brain, liver and kidney obtained from control and PS-NP-exposed immature rats: MDA concentration **(A–C)**; carbonyl groups content (CGC) **(D–F)**; 8-OHdG **(G–I)**. Data are presented as means ± SD from 6 control and 10 PS-NPs treated animal samples; *p < 0.05; **p < 0.01 (Unpaired t-test).

Carbonyl group content (CGC), measured as a marker of irreversible protein oxidation, was found to be significantly higher in all tissues collected from PS-NP-exposed animals (p < 0.01 vs. controls) (Figures 7D–F), indicating that PS-NP-induced oxidative damage mainly affects cellular proteins but not membrane lipids. The level of 8-OHdG, which is a key biomarker of oxidative DNA damage caused by hydroxyl radical attack on guanine bases, did not change in the tissues of exposed animals compared to the control group (Figures 7G–I), although an increased trend was observed in the liver. The results clearly indicate the presence of oxidative stress-induced damage to key cellular macromolecules, pointing to the liver as the main organ in which proteins and lipids are affected by PS-NPs exposure.

Since oxidative stress occurs when excess free radicals are not properly balanced by defense mechanisms, we examined the effectiveness of antioxidant systems in our experimental conditions. To assess the tissue-specific potential of the major non-enzymatic antioxidant, we measured the concentration of reduced glutathione, which was unchanged in the brain and liver but significantly increased (p = 0.0389) in the kidney of exposed animals (Figures 8A–C). In turn, total glutathione concentration (GSH + GSSG) increased in brain (p = 0.0321) but remained unchanged in other tissues examined (Figures 8D–F).

**FIGURE 8:**
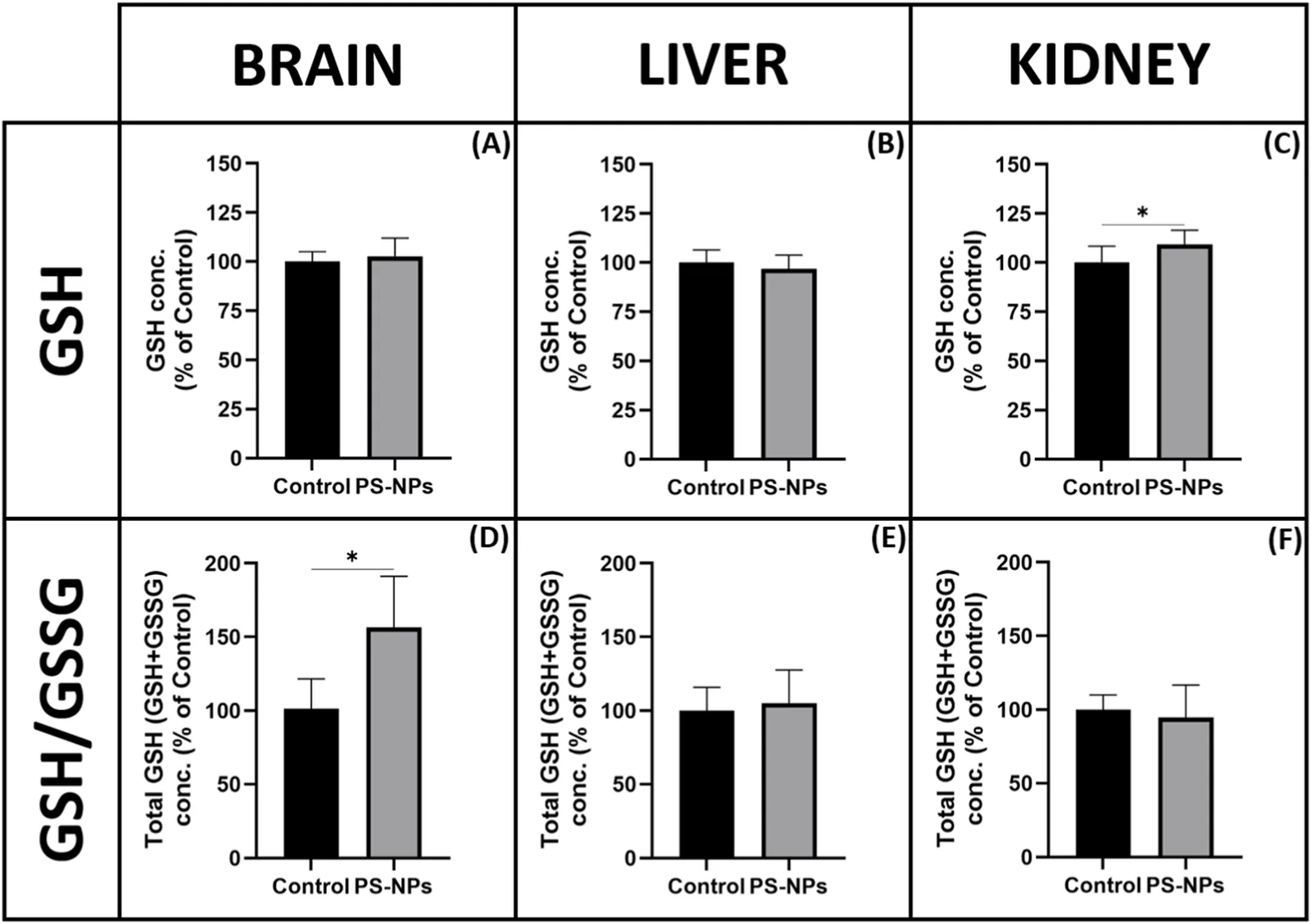
Reduced glutathione (GSH) **(A–C)** and total glutathione (GSH/GSSG) concentration **(D–F)** in the brain **(A,D)**, liver **(B,E)**, and kidney **(C,F)** isolated from control and PS-NP-exposed immature rats. Data are presented as means ± SD from 6 control and 10 PS-NPs treated animals; *p < 0.05 (Unpaired t-test).

Since the total antioxidant capacity also consists of antioxidant enzymatic systems, we next examined the status of selected enzymes, such as catalase (CAT), superoxide dismutases (SOD1 and SOD2) and glutathione peroxidase (GSH-Px4) (Figure 9). The activity of CAT (Figure 9A.1a,d,g) and SODs (Figure 9B.1a,f,k) did not change. GSH-Px was the only enzyme whose activity increased significantly in all tissues tested (Figure 9C.1 a,d,g; p < 0.05). However, at the transcriptional level, the expression of both *Cat* and *Sod1* mRNAs increased significantly in the kidney (p = 0.0027 and p = 0.0008, respectively) and brain (p < 0.05, both) but decreased significantly in the liver compared to control group (p = 0.0430 for *Cat*; p = 0.0077 for *Sod1*). In contrast, *Sod2* mRNA expression was elevated only in the kidney (p = 0.0027) with no changes in other tissues. Expression of the *Gsh-Px4* isoform was upregulated in the kidney (p = 0.0013) and brain (p = 0.0494) but was reduced in the liver (p = 0.0132). At the protein level, we observed similar trends of changes as for mRNAs, with the exception of GSH-Px4, whose protein expression remained unchanged in the brain, although it increased at the transcriptional level. Overall, the results highlight differences in susceptibility to oxidative stress, identifying the liver as the organ most affected upon exposure to PS-NPs. The profiles of changes in both the oxidative stress markers and antioxidant response of individual tissues are summarized in Table 1.

**FIGURE 9:**
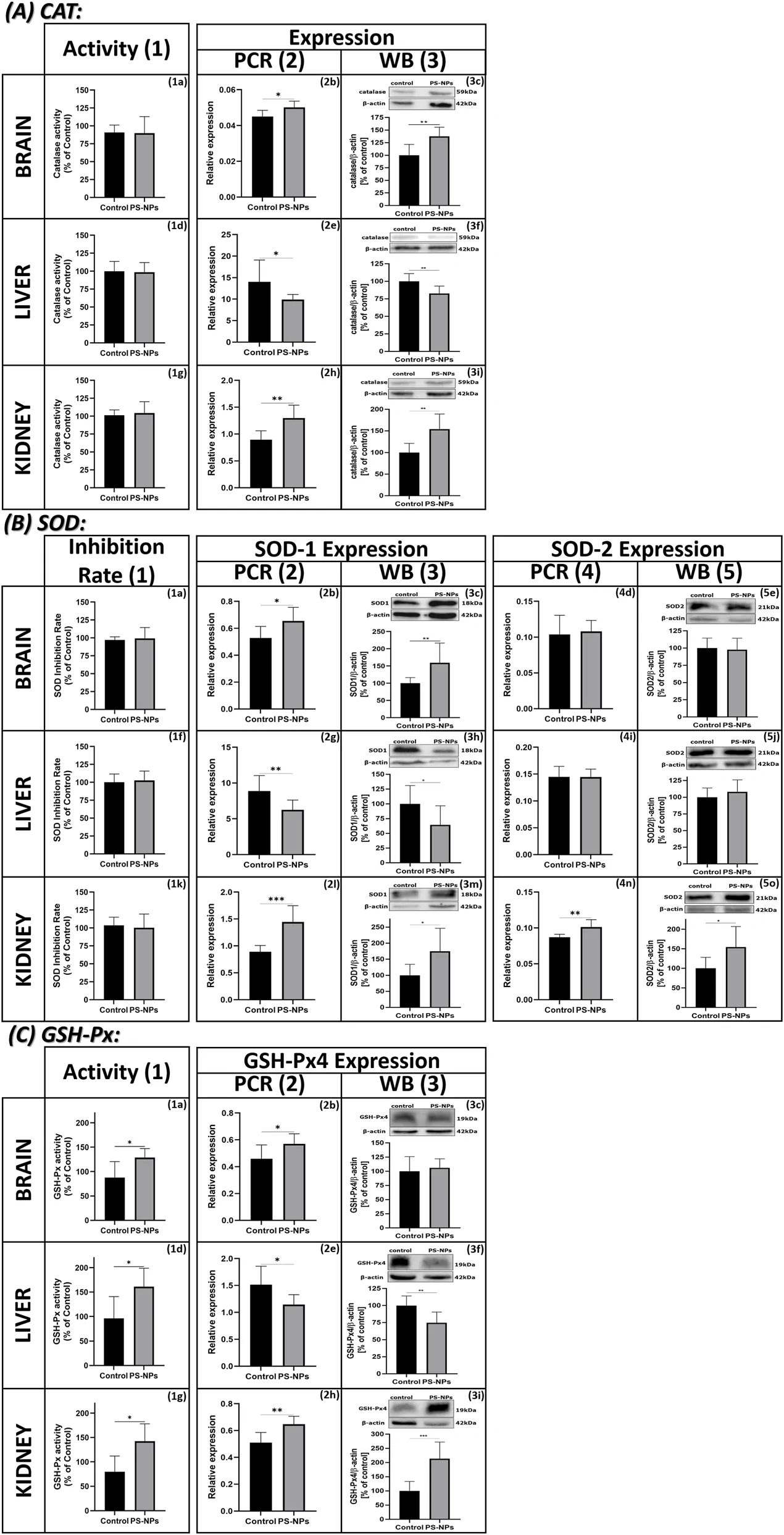
Activity and expression of major antioxidant enzymes in tissues isolated from control and PS-NPs-exposed immature rats. The panel includes CAT **(A)**, SOD **(B)**, and GSH-Px **(C)**. Enzyme activity (inhibition rate in the case of SOD) (1). Relative expression of enzymes at the mRNA (2 and 4), and protein (3 and 5) levels shown in graphs and representative immunoblots. Data are presented as means ± SD from 6 control and 10 PS-NPs treated independent experiments using separate animal samples; *p < 0.05; **p < 0.01; ***p < 0.001 (Unpaired t-test and Mann-Whitney test for B3c; B4n; C1a).

**TABLE 1:** The profile of changes in the oxidative stress markers and antioxidant defense systems in tissues of the PS-NP-exposed rats compared to the controls.

Parameter			Brain	Liver	Kidney
OS Markers		MDA	UC	↑	UC
		CGC	↑	↑	↑
		8-OHdG	UC	UC	UC
Antioxidant Systems	Non-Enzymatic	GSH	UC	UC	↑
		GSH/GSSG	↑	UC	UC
	Enzymatic	CAT: Activity PCR W-B	UC ↑ ↑	UC ↓ ↓	UC ↑ ↑
		SOD: ACTIVITY (total) PCR – SOD1 SOD2 W-B – SOD1 SOD2	UC ↑ UC ↑ UC	UC ↓ UC ↓ UC	UC ↑ ↑ ↑ ↑
		GSH-Px4 Activity PCR W-B	↑ ↑ UC	↑ ↓ ↓	↑ ↑ ↑

The table summarizes statistically significant changes between control and exposed groups in the respective parameters of oxidative stress (OS) markers (MDA, CGC, 8-OHdG) and non-enzymatic (GSH, GSH/GSSG) and enzymatic (CAT, SOD1, SOD2, GSH-Px4) antioxidant systems presented above on Figures 7–9; ↑ - increase; ↓ - decrease; UC, unchanged vs. control.

## 4 Discussion

### 4.1 Nanoplastics overcome biological barriers in immature organisms during prolonged exposure

As the world struggles with the growing problem of plastic pollution, thorough studies are needed to investigate the potential health concerns resulting from MPs/NPs originating from the gradual fragmentation of plastic waste. Importantly, modeling of developmental toxicity, and neurotoxicity of MPs/NPs in particular, should be conducted at levels relevant to real-world scenarios.

In this study, PS-NPs were administered daily at an environmentally plausible dose calculated based on available literature data. These data indicate that human ingestion of MPs/NPs through different exposure routes was predicted to range from 0.1 to 5 g per week (Senathirajah et al., 2021), corresponding to 1.4–71.4 mg/kg body weight (b.w.) in a 70 kg person, while the daily intake of MPs/NPs was estimated to be 2.4–700 mg (Priya et al., 2022).

Exposure to MPs/NPs may occur via multiple environmental routes, including food, water, and daily-life products that release plastic particles. Recently, the average total concentration of detectable plastic particles in human blood was reported to be at the level of 1.6 μg/mL (Leslie et al., 2022). Given that the total blood volume of a 70 kg healthy adult is approximately 4900 mL, the calculated total amount of plastic particles in the bloodstream is 7840 μg, corresponding to an ingested dose of 112 μg/kg b.w. (Du et al., 2024).

Taking into account that the estimated bioavailability of plastic particles ranges between 0.2% and 1.7% of the exposure dose (Walczak et al., 2015a), the default amount of administered plastic particles is 11.2 mg/kg b.w. (assuming an absorption rate of 1%) in an adult individual, and is respectively lower in a young individual. It could therefore be assumed that our calculated dose of 1 mg/kg was within the range of possible actual absorbed doses.

It is worth noting that measuring plastic particles in complex biological matrices is challenging because established analytical methods are not sufficiently adapted or validated, and present various limitations (Paul et al., 2020). This indicates that it is difficult to define what actually constitutes an “environmentally relevant dose”.

To our knowledge, this study is among the first to compare the multisystemic prooxidant effects of nanoplastics in immature rats. Previous *in vivo* studies have mostly focused on the toxicity of PS-NPs in adult organisms, paying little attention to young, developing individuals. However, it is known that immature organisms are extremely susceptible to toxic insults due to biological determinants, such as anatomical, physiological, biochemical, and behavioral factors, which enhance the absorption of various toxicants, thereby increasing the health risks of exposure (Strużyńska et al., 2022). Therefore, the toxic effects of PS-NPs in a young animal model need to be evaluated.

Experimental data have indicated that developing organs exhibit lower functionality, which increases the intake and toxicity of various toxic agents, including nanoparticles (Yong et al., 2020). For example, the immature intestine expresses specific transport mechanisms that allow for excessive absorption of various substances from the gastro-intestinal tract (Bearer, 1995), which may also be responsible for the increased uptake of NPs (Dąbrowska-Bouta et al., 2021), including PS-NPs. In addition, a lower glomerular filtration rate in the developing kidney is responsible for the lower excretion of toxic metabolites (John, E. G, 1991).

Our results revealed that exposure to a relatively low dose of PS-NPs by oral gavage results in the presence of PS-NP-derived red fluorescence in all examined tissues. This indicates that they possess the ability to cross biological barriers, such as the gut-blood barrier (GBB) and the BBB. After being absorbed in the intestine, they are transported by the circulatory system and accumulate in the examined organs, including the liver, kidney and brain. The study by Değermenci’s (Değermenci et al., 2025) also confirmed the presence of PS-NPs in testicular tissue, indicating that the blood-testis barrier does not pose an obstacle to these NPs.

Although fluorescence tracking (IVIS and confocal microscopy) provides clear qualitative evidence of tissue distribution, fluorescence intensity should be considered a semi-quantitative index of retention rather than a direct measure of PS-NPs concentration. The accumulation rate of PS-NPs estimated based on the intensity of fluorescence signals in both *ex vivo* imaging using the IVIS system and immunohistochemical (IHC) analysis, is highest in the liver and the kidney, and lowest in the brain. This tissue profile of PS-NPs biodistribution is similar to that reported by (Deng et al., 2017), who found that MPs (5 and 20 µm) accumulated in the liver, kidney and gut of exposed adult mice, with a distribution pattern strongly dependent on the size of MPs. The small size of MPs/NPs allows them to penetrate deeply into organs, underscoring its influence on the biodistribution profile. According to a previous study, smaller PS-NPs (130 nm), as opposed to larger ones (1200 nm), exhibited prolonged retention, predominantly in the liver and spleen, impacting liver function (Jing et al., 2023). Also, in the study of (Yong et al., 2020), ingested MPs/NPs could mainly be found in the gut, liver and kidney of mice.

Results from (Hou et al., 2024), utilizing a magnetic resonance imaging (MRI)-based tracking method, confirmed that the liver is the primary accumulation site of PS-MPs functionalized with Fe_3_O_4._ Furthermore (Du et al., 2024), demonstrated high accumulation of PS-MPs/NPs (50, 100, 500 nm) in the intestine and spleen, showing a dose-dependent effect. The liver and the heart showed lower fluorescence, while only the smallest particles (50 nm) were detected in the kidneys and testes. No particles were observed in the brain or lungs, indicating a size-dependent pattern of biodistribution. On the other hand, even larger PS-NPs (293 nm) administered orally to mice were detected in brain tissue within 2 h of exposure, indicating rapid uptake (Kopatz et al., 2023), which contradicts the result of Du et al. (2024).

Under our experimental conditions, the immature brain is an important target for small (25 nm) PS-NPs, although they accumulate in this organ with the lowest efficiency compared to other tissues. We confirmed their presence in both astrocytes and neurons, in agreement with our previous *in vitro* studies, which proved capable of PS-NPs internalization by these cell types (Adamiak et al., 2025).

The free passage of PS-NPs across the BBB, whose physiological role is to tightly regulate the bidirectional transport of substances between the blood and the brain, is particularly disturbing in the context of PS-NPs neurotoxicity.

The mechanisms of this transvascular passage are not yet established. However, the “immaturity” of the BBB may be responsible for the increased vulnerability of the developing CNS to various toxicants. Unique and functionally much more effective transport mechanisms operating at the level of the BBB, such as over-efficient transport of certain amino acids (Ek et al., 2012), have been identified as a common mechanism underlying developmental neurotoxicity due to the intensive co-transport of various toxic substances.

### 4.2 Ultrastructural and molecular characteristics indicative of PS-NP-induced oxidative stress in various organs of exposed rats

Previous studies have shown that both NPs and MPs can pass the gut barrier and accumulate in the liver of exposed animals in amounts closely related to their size, producing toxic effects, such as changes in glucose and amino acid metabolism, and morphological alterations in the form of hepatocyte hypertrophy and necrosis (for a review see (Yin et al., 2022)). Similarly, our previous study indicated that PS-NPs administered to immature rats exposed at a dose of 1 mg/kg b.w. for 3 weeks passed the gut barrier and accumulated in the epithelium and subepithelial layer of the intestine, simultaneously inducing changes in the expression of genes related to gut barrier integrity, nutrient absorption and endocrine function (Augustyniak et al., 2025). Moreover, we also identified changes in the metabolite profiles in the intestine extracts of exposed rats that were consistent with the observed molecular markers of inflammation and oxidative stress. However, there are also contradictory data showing that exposure to PS-NPs (0.2, 1, or 10 mg/kg PS-NPs for 30 days) did not cause pathological changes in the liver, lung or brain cortex of young mice (4 week-old mice with a mature BBB) (Xiao et al., 2022).

Our current results revealed that exposure of immature rats to a low dose (1 mg/kg b.w.) of PS-NPs leads to their significant deposition in the tested organs, particularly in the liver and, to a lesser extent in the kidney and brain, suggesting that immaturity promotes significant deposition of PS-NPs in tissues thereby increasing the risk of adverse consequences over time of exposure. Therefore, morphological, molecular and biochemical analyses were performed to assess the effects of PS-NPs on these tissues.

First, we observed significant alterations in the ultrastructure of mitochondria, organelles that are crucial for maintaining cellular homeostasis, regulating energy status and apoptotic processes, constituting a key interface in the brain’s response to various environmental stimuli. Mitochondria are typically slightly elongated in shape and are limited by an outer membrane and a complex inner membrane that forms tightly packed infoldings (cristae), functionally necessary to increase the surface area for ATP production (Freya and Mannellab, 2000).

In our study, this normal structure was frequently disrupted in the brain and, to a lesser extent, in the kidney and liver, where mitochondria showed vacuolization, swelling, cristae destruction, and outer membrane disruption. This morphological degradation, coupled with hepatic glycogen depletion, indicates mitochondrial dysfunction and confirms that dysregulated mitochondria lose their capacity for efficient ATP production. These results are consistent with the study by (Seward et al., 2025), who showed that 50 nm PS-NPs can impair both synaptic and non-synaptic mitochondrial complexes by selectively impairing electron transfer between complexes I–III and II–III, and inhibiting brain mitochondrial respiration.

Exposure to PS-NPs can also disrupt mitochondrial dynamics across tissues examined, which is associated with balanced processes of mitochondrial fission and fusion necessary for cell survival, adaptation to metabolic changes, and removal of damaged mitochondria (Youle and Van Der Bliek, 2012). As we have seen, the division process predominates in the liver and kidney, which is a classic response to cellular stress, indicating an attempt to isolate damaged organelle fragments, while in the brain, a clear shift in mitochondrial dynamic towards elongated or interconnected networks was observed.

Ultrastructural observations indicate that disruption of mitochondrial dynamics may be a pathophysiological mechanism induced by PS-NPs in immature rats. However, this issue requires further molecular studies to confirm the presence of fusion/fission-specific proteins.

One of the critical consequences of mitochondria damage and dysfunction is the generation of reactive oxygen species (ROS) leading to oxidative stress. The adverse effects of oxidative stress resulting from excessive ROS production include lipid peroxidation in cell membranes (measured by MDA), irreversible protein oxidation and DNA damage. Under our experimental conditions, we identified a statistically significant enhancement of MDA levels only in the liver of PS-NP-exposed rats, while the content of carbonyl groups increased significantly in all examined tissues, indicating oxidative damage mainly to proteins (Figure 7; Table 1).

This suggests that the oxidative stress induced by PS-NPs is rather moderate, with proteins acting as a primary target protecting the cell from PS-NP toxicity. PS-NPs accumulate mainly in the cytoplasm, lysosomes, and extracellular space. ROS generated there affect cytoplasmic and structural proteins before they can reach the nucleus and damage DNA, which explains the lack of 8-OHdG marker. This phenomenon aligns with the findings of Dahl et al. (2015), who identified the proteome as the major intracellular target of ROS. Furthermore, as suggested by others (Dalle-Donne et al., 2003), protein carbonylation serves as an early, sensitive and stable marker of damage, frequently manifesting before the onset of the lipid peroxidation cascade or nuclear DNA damage. In the context of organ specificity, in the case of the brain, the susceptibility of proteins in the absence of DNA damage (8-OHdG) has been well documented by Martínez-Orgado et al. (2023), who found that nervous tissue (especially during development or after exposure to xenobiotics) initiates protein carbonylation before neuronal death or severe genomic damage, confirming the role of proteins as a “ROS buffer”.

There was no massive destruction of membrane structures in the brain and kidney (no increase in MDA level), which is consistent with the increased activity and overexpression of GSH-Px - the enzymatic antioxidant system of the lipid phase - in these tissues. The opposite situation was observed in the liver, where downregulation of GSH-Px4 was noted with increasing MDA concentration.

This strongest liver reaction among the organs examined is due to its detoxification functions. However, when overloaded, its capacity is no longer sufficient to counteract the harmful effects of PS-NPs. In our study, this organ exhibited the strongest PS-NPs fluorescence, suggesting that this threshold may have been exceeded. In contrast, the brain is less resistant to the effects of xenobiotics, so even smaller aggregates may contribute to the development of pathological changes.

The contribution of oxidative stress to plastic-induced cell death was previously confirmed by (Liang et al., 2021), who showed that the combined toxicity of both PS-MPs and PS-NPs on intestinal barrier dysfunction in mice was primarily caused by ROS-mediated epithelial cell apoptosis. Similarly, the toxic effects of MPs and NPs in the liver of marine species were induced by the overproduction of ROS and subsequent development of oxidative stress and inflammation (Li et al., 2020). In contrast, no significant alterations were observed in oxidative stress-related markers in the liver, lung, intestine, cerebral cortex or serum of adult mice treated with 0, 0.2, 1, or 10 mg/kg PS-NPs for 30 days, although the expressions of genes related to mucus secretion were altered, as was the composition of the gut microbiota (Xiao et al., 2022).

Discrepancies between the results may be due to several experimental variables, such as the age and developmental stage of the animals, as well as the particle size and surface functionalization. The PS-NPs used in our study were characterized by extremely small sizes (25 nm), a factor indicated by many researchers as the cause of increased size-dependent toxicity (Dong et al., 2020). In addition, they are covalently coated with an amino group, which imparts a strongly negative zeta potential. This enhances the particles’ stability (Adamiak et al., 2025) in solution and improves their absorption within the body. Many authors have also highlighted the influence of the functional group type on NP toxicity, noting that amino groups are associated with increased toxic potential (Banerjee et al., 2021). The animals were exposed to PS-NPs via the oral route, the most common route of exposure in humans, which determined the choice of this particular method. Another factor contributing to the increased toxic potential of PS-NPs in our study is a developmental stage of the experimental animals, in which various physiological barriers (including the BBB) are not yet fully developed (Utsumi et al., 2000).

### 4.3 Tissue-specific differences in the profiles of antioxidant defense systems

Under physiological conditions, the effective action of enzymatic and non-enzymatic antioxidant defense systems prevents damage to molecules. Oxidative stress develops when excess ROS are not counterbalanced by cellular antioxidant mechanisms. Therefore, the increase in the levels of oxidative stress markers we observed clearly indicates a reduced effectiveness of antioxidant systems in removing ROS, which results in increased oxidative damage to lipids and proteins.

This study provides a comparative analysis of the antioxidant response in different tissues of immature rats exposed to a low dose of PS-NPs. The non-enzymatic GSH-based system was induced only in the kidney, but not in the liver and brain. Furthermore, the activity of enzyme-based antioxidant defense systems, which constitute the first line of defense against superoxide and hydrogen peroxide, was not significantly elevated, with the exception of GSH-Px activity in all tissues examined. However, activation of the cellular transcriptional/translational machinery was observed, particularly in the kidney, where all enzymes, CAT, SOD1, SOD2, GSH-Px4, were upregulated. In brain tissue, these mechanisms were less effective, although deposition of PS-NPs was lower compared to the kidney. No increased GSH content was found, and *Sod2* expression was not activated, nor was the expression of GSH-Px4 at the protein level. In addition, the observed morphological abnormalities in mitochondria were consistent with the lack of effective activation of *Sod2* expression, which is the mitochondrial isoform of the enzyme.

While CAT and SOD1 showed organ-specific transcriptional and translational shifts (upregulation in brain and kidney; downregulation in liver), the total functional activity of the enzyme remained unchanged compared to the control group. This discrepancy can be attributed to post-translational modifications, functional enzyme saturation, or partial oxidative inactivation of existing enzyme molecules by ROS, which prompts a compensatory increase in gene transcription without increasing net catalytic efficiency. In turn, GSH-Px4 demonstrated coordinated induction at the functional activity, mRNA, and protein levels in the kidney.

Interestingly, in the brain tissue of PS-NP-exposed rats, GSH levels remained unchanged while total glutathione (GSH + GSSG) levels increased significantly. This pattern suggests a potential accumulation of oxidized glutathione (GSSG) and a shift toward a more oxidized intracellular redox environment, further pointing to the vulnerability of the immature brain. However, as GSH and total glutathione were evaluated using separate assays, dedicated unified GSSG quantification will be valuable to further confirm this shift in redox balance.

While certain compensatory mechanisms were activated in the brain and especially in the kidney, analysis of changes in the liver indicates its particular susceptibility to oxidative stress, manifested by a decrease in the expression of antioxidant genes. Despite the initial stability of GSH levels and overall enzymatic activity, suppression of *Cat*, *Sod-1* and *Gsh-Px4* at the transcriptional level suggests a failure of liver defense mechanisms. These results identify the liver as the organ most affected by the exposure to PS-NPs, distinguishing it from the kidney. In addition to the liver, the immature brain also appears to be vulnerable to the adverse effects of PS-NPs if the results are considered in the context of organ-specific accumulation rate. Indeed, the developing brain has been recognized as highly susceptible to oxidative stress due to its high rate of oxygen consumption and the large number of glutamatergic NMDA receptors (Ikonomidou and Kaindl, 2011) combined with the low efficiency of enzymatic antioxidant defense systems (Shim and Kim, 2013).

## 5 Conclusion

The results revealed that after ingestion at a low dose of 1 mg/kg b.w., small PS-NPs (25 nm) overcome biological barriers and accumulate in the tested organs, such as the liver, kidney and brain. Despite this low level of exposure, absorption of PS-NPs may lead to a systemic, toxicologically significant effect, namely, oxidative stress.

It can be concluded that under our experimental conditions, the liver is particularly vulnerable to the pro-oxidant potential of PS-NPs. This effect is presumably due to the much greater deposition of PS-NPs in the liver compared to the kidney and brain. However, brain tissue, despite a much lower concentration of deposited PS-NPs compared to the kidney, is characterized by a less effective antioxidant response, making it a potential target for harmful effects. Importantly, there is no data on whether and to what extent brain tissue is able to clear itself of PS-NPs. Previous studies on the long-term effects of silver NPs (AgNPs) after early postnatal exposure revealed that silver was still present in the rat brain, although its concentration in other tissue compartments decreased concomitantly over time (Dąbrowska-Bouta et al., 2021). This suggests that the immature brain may be a target for long-term toxicity of nanoparticles, including PS-NPs.

## 6 Limitations of the study

While this study provides novel insights into the systemic distribution, prooxidant potential, and mitochondrial toxicity of low-dose nanoplastics in developing rats, several methodological limitations should be acknowledged when interpreting and generalizing our findings.

A total of 36 immature rats were utilized in this study (15 control animals and 21 PS-NPs-treated animals). Sample size determination was guided by G Power calculations specifically for the primary quantitative biochemical and molecular assays. This calculation should not be interpreted as requiring n = 6 for every single experimental procedure. To strictly adhere to the ethical requirements set by the Local Ethics Committee and minimize total animal usage (3Rs principles), qualitative and morphological imaging procedures (IVIS *ex vivo* imaging, confocal microscopy, and TEM) were performed on dedicated separate subsets of animals. Consequently, sample sizes vary across experimental endpoints, which may represent methodological limitation of the study design.

Due to restrictive regulations set by the Local Ethics Committee for Animal Experimentation aimed at minimizing total animal sacrifice, a relatively small number of independent litters (three mothers) was utilized. Offspring originating from the same mother share a common maternal background and may not represent entirely independent biological observations. To mitigate this potential confounder within ethical constraints, we adopted a “Split-Litter Design” as a recommended randomization strategy. Pups from each of the three litters were equally distributed across the control and PS-NP-treated groups, allowing each litter to serve as its own internal control. Nevertheless, maternal effects were not explicitly included as a separate factor in the primary statistical model, and this should be taken into consideration.

Reduced glutathione (GSH) and total glutathione (GSH + GSSG) levels were quantified using separate commercial assays normalized to their respective controls. Consequently, the expansion of the oxidized glutathione (GSSG) pool in brain tissue was inferred from elevated total glutathione alongside stable GSH levels, rather than directly measured via a single integrated enzymatic recycling assay.

Finally, the experimental paradigm focused on a single low, environmentally plausible dose (1 mg/kg b.w./day) administered during a specific developmental window (PND 14–35). Multi-dose response curves and long-term post-exposure recovery dynamics were beyond the scope of the present study and warrant future investigation.

## Data Availability

The datasets presented in this study can be found in online repositories. The names of the repository/repositories and accession number(s) can be found below: https://doi.org/10.18150/MJR1VD, RepOD.
